# Identification of four novel hub genes as monitoring biomarkers for colorectal cancer

**DOI:** 10.1186/s41065-021-00216-7

**Published:** 2022-01-29

**Authors:** Danqing Luo, Jing Yang, Junji Liu, Xia Yong, Zhimin Wang

**Affiliations:** 1 Spinal Surgery, The Second Affiliated Hospital, Hengyang Medical School, University of South China, No.35 Jiefang Avenue, Hengyang, 421000 China; 2 Anorectal Department, The Second Affiliated Hospital, Hengyang Medical School, University of South China, No.35 Jiefang Avenue, Hengyang, 421000 China; 3 Department of Oncology, The Central Hospital of Shaoyang, No. 36 Qianyuan Lane, Hongqi Road, Shaoyang, 422000 China; 4 Biomed Center, Wuhan Institute of Technology, Wuhan, 430000 China; 5 Human Resources Department, The Second Affiliated Hospital, Hengyang Medical School, University of South China, No.35 Jiefang Avenue, Hengyang, 421000 China

**Keywords:** Colorectal Cancer, Potential Pathogenesis, Robust Rank Aggregation, Weighted Gene Co-expression Network Analysis, Hub Gene

## Abstract

**Background:**

It must be admitted that the incidence of colorectal cancer (CRC) was on the rise all over the world, but the related treatment had not caught up. Further research on the underlying pathogenesis of CRC was conducive to improving the survival status of current CRC patients.

**Methods:**

Differentially expressed genes (DEGs) screening were conducted based on “limma” and “RobustRankAggreg” package of R software. Weighted gene co-expression network analysis (WGCNA) was performed in the integrated DEGs that from The Cancer Genome Atlas (TCGA), and all samples of validation were from Gene Expression Omnlbus (GEO) dataset.

**Results:**

The terms obtained in the functional annotation for primary DEGs indicated that they were associated with CRC. The MEyellow stand out whereby showed the significant correlation with clinical feature (disease), and 4 hub genes, including ABCC13, AMPD1, SCNN1B and TMIGD1, were identified in yellow module. Nine datasets from Gene Expression Omnibus database confirmed these four genes were significantly down-regulated and the survival estimates for the low-expression group of these genes were lower than for the high-expression group in Kaplan-Meier survival analysis section. MEXPRESS suggested that down-regulation of some top hub genes may be caused by hypermethylation. Receiver operating characteristic curves indicated that these genes had certain diagnostic efficacy. Moreover, tumor-infiltrating immune cells and gene set enrichment analysis for hub genes suggested that there were some associations between these genes and the pathogenesis of CRC.

**Conclusion:**

This study identified modules that were significantly associated with CRC, four novel hub genes, and further analysis of these genes. This may provide a little new insights and directions into the potential pathogenesis of CRC.

**Supplementary Information:**

The online version contains supplementary material available at 10.1186/s41065-021-00216-7.

## Introduction

Colorectal cancer (CRC) with the second highest cancer-related mortality rate, was the third most common cancer in men and the second most common in women worldwide, caused approximate 881,000 deaths worldwide in 2018 [1]. Gene mutation, epigenetic changes including cytosine guanine (CpG) island methylator phenotype (CIMP) [2] and environmental factors including diet, sedentary lifestyle and gut microbiota played a vital role in the progression of CRC [3]. It’s worth mentioning that obesity was an important factor, a meta-analysis showed that 33% increase in the risk for an obese person compared with a person of normal weight, which may be mediated by insulin resistance [3]. 75–80% cases were sporadic, with hereditary factors contributing to 20–25% of CRC [4]. Therefore, we could consider that the complex interaction between susceptibility genes and environmental factors was the main reason for the occurrence and development of CRC. Early CRC symptoms were not obvious, most patients with significant symptoms were often advanced CRC. Metastases were common in advanced CRC, and the survival rate for patients with metastases is only about 14% [5]. Therefore, we need to make efforts to study the potential pathogenesis of CRC to find new screening and early detection methods. Numerous studies have focused on biomarkers associated with CRC including susceptibility genes and deoxyribonucleic acid (DNA) methylome. Bagheri et al. [6] believed that tissue factor pathway inhibitor 2 (TFPI2) and N-myc downregulated gene family (NDRG) 4 with high enough sensitivity and specificity were nominated as the new CRC screening gene in peripheral blood mononuclear cells. In addition, Manoochehri’s study [7] revealed three-gene, including NGFR, FGF2, and PROM1 genes, signatures as potential therapeutic targets and also candidate molecular markers in CRC chemoradioresistance. In a prospective targeted sequencing involving 1134 CRC samples, mutations in adenomatous polyposis coli (APC) and CTNNB1 genes were identified, which increased oncogenic WNT pathway changes to 96% of CRCs [8]. As a key driver of tissue stem cell types, WNT signaling pathway involved in both the development and homeostasis of tissues [9]. Mutations in WNT signaling pathway components lead to a variety of growth diseases and cancers, including CRC, and many researches about therapeutic approaches to target the WNT pathway (especially Wnt/β-catenin signaling pathway) and their clinical applications were reported. Deletion of CRC gene expression may be associated with hypermethylation of CpG islands in some susceptible genes [10], and CIMP in subtype of CRC was reported [2].

Current diagnostic methods of CRC are based on histopathologic examination, while treatment plans and prognostic predictions usually refer to the tumour, node and metastasis (TNM) stage, which was first mentioned in 1968 [11]. It was well known that heterogeneous genomes of CRC patients can provide important prognostic information. It was a future development trend to conduct individualized treatment for patients with heterogeneous genes, so it was necessary to further understand the underlying pathogenesis of CRC and establish specific CRC gene blueprint. Sun’s study [12] identified that seven key genes, including PPBP, CCL28, CXCL12, INSL5, CXCL3, CXCL10 and CXCL11, were identified as important molecular markers, contributing to the screening, diagnosis, prognosis and new therapeutic targets of CRC. In addition, TIMP1, LZTS3, AXIN2, CXCL1, ITLN1, CPT2 and CLDN23 genes have also been confirmed to be related to the pathogenesis of CRC [13]. In terms of sensitive medicine screening, a study [14] confirmed that the combination of gefeitinib and regorafenib in the treatment of HCT116, CT26 and SW948 colorectal cancer cell lines may be a promising strategy for the treatment of colorectal cancer. At present, a large number of key genes had been identified related to the pathogenesis of CRC, which also brings more possibilities for the gene identification and diagnosis of CRC. Based on the uncertain possibilities of mechanics for CRC, this study will conduct the identification of genes closely related to CRC, which might provide some new insights for future individualized and comprehensive therapy.

## Materials and methods

### Microarray detection and differential expression analysis

Nine eligible microarrays from GEO dataset, including GSE4183 [15], GSE44076 [16, 17], GSE23878 [18], GSE32323 [19], GSE110223 [20], GSE110224 [20], GSE33113 [21, 22], GSE37364 [23], and GSE9348 [24], were enrolled in the study. The relevant information of the 9 datasets included in the study was list in Table 1. Screening of DEGs can identify the differences in gene expression levels between tumor tissues and matched normal tissues and identify the specific genes correlated with biological characteristics in tumors. We employed the edgeR package [25] of R software (Version 3.6.3) to analyze the differences between non-malignant samples and colon adenocarcinoma (COAD) tissues in the TCGA-COAD dataset. The statistical significance of genes between datasets was assessed by a linear model implemented in the “limma” package [26]. Differentially expressed genes (DEGs), including significantly downregulated and upregulated genes, were selected for further study with the cut-off criteria of false discovery rate (FDR) < 0.05 and |log_2_ fold change (FC)| >1. *P*-value threshold <0.05 was set as statistically significant for DEGs among the genes. The process of DEGs sorting by *P*-value using robust rank aggregation (RRA) method [27] was carried out. The integrated DEGs were used for subsequent analysis.

**Table 1:** Characteristics of the data sets enrolled in the study

Dataset ID	Number of carcinoma samples	Number of normal samples	Country	GPL ID
GSE4183	15	8	Hungary	GPL570
GSE44076	98	148	Spain	GPL13667
GSE23878	35	20	Saudi Arabia	GPL570
GSE32323	17	17	Japan	GPL570
GSE110224	17	17	Greece	GPL570
GSE110223	13	13	Greece	GPL96
GSE33113	90	6	Netherlands	GPL570
GSE37364	14	38	Hungary	GPL570
GSE9348	70	12	Singapore	GPL570

### Visualization of gene expression patterns and chromosome locations

In the section, top 100 DEGs including top 50 up-regulated genes and top 50 down-regulated genes were uploaded to the National Center for Biotechnology Information Gene for chromosomal locations. Then, visualization of the expression patterns and chromosomal locations were conducted in “OmicCircos” package [28].

### Functional annotation and visualization

Gene Ontology (GO) and Kyoto Encyclopedia of Genes and Genomes (KEGG) pathway enrichment analysis were performed for the top 300 DEGs found in the integrated DEGs with Database for Annotation, Visualization and Integrated Discovery (DAVID) [29]. Functional annotation and enrichment pathways were visualized in “GOplot” package [30].

### Weighted gene co-expression network analysis

Weighted gene co-expression network analysis (WGCNA) was performed in the integrated DEGs that from The Cancer Genome Atlas (TCGA). Nine samples were outliers with the threshold for identifying outlier sample was set as −0.25 (Supplemental Fig. 1). Correlation matrix (gene expression matrix), which each row represents a different gene and each column represent a sample, was formed. The correlation matrix was transformed to an adjacency matrix based on proper soft-thresholding parameter β, which can enhance high correlations and weaken low correlations. Then, modules with the mini-size of module gene numbers set as 30 were obtained after an average linkage hierarchical clustering was carried out based on topological overlap matrix (TOM) dissimilarity measure [31]. In current study, β = 9 was chosen to pledge a scale-free network (Supplemental Fig. 2). Module eigengene (ME) was the first principal component obtained from the principal component analysis of the expression matrix of each gene, and interesting module was identified by calculating the relevance between MEs and clinical features. Furthermore, two parameters were calculated: the Pearson’s correlation between the ME of each module and clinical information was defined as module significance (MS), the log10 transformation of the *P*-value was defined as gene significance (GS). The functional annotation and enrichment analysis were restricted to KEGG and GO in DAVID [29].

### Identification, validation and efficacy evaluation for hub genes

Hub genes were defined on genemodumemberships >0.80, and genetics significance >0.20. All samples of validation were from GEO dataset, including GSE4183 [15], GSE44076 [16, 17], GSE23878 [18], GSE32323 [19], GSE110223 [20], GSE110224 [20], GSE33113 [21, 22], GSE37364 [23], and GSE9348 [24]. Evaluation of diagnostic efficacy was typically based on the summary index of receiver operating characteristic (ROC) curve and the area under curve (AUC). Therefore, ROC curve that as an effective method of evaluating the performance of diagnostic tests was plotted and AUC was calculated with “pROC” package [32] to evaluate the diagnostic performance for hub genes.

### Kaplan-Meier survival analysis

In the section, patients were divided into two groups according to median expression value of each hub genes to plot survival curve. Survival curves constructed under the control of a single variable for a hub gene were compared using log-rank test. Log-rank test could indicate that whether there was statistical significance between two groups. Survival analysis was conducted in Gene Expression Profiling Interactive Analysis (GEPIA) [33].

### DNA methylation analysis of hub genes

DNA methylation, the addition of a methyl group to the carbon 5-position of cytosine within a CpG dinucleotide, was a common and early event in cancer [34]. DNA methylation was increasingly being incorporated in biomarker studies because of its potential prognostic value. In current study, DNA methylation datum of hub genes obtained from the human disease methylation database version 2.0 [35]. Furthermore, the visualize DNA methylation, expression and clinical data (MEXPRESS) [36] guided the relationship between hub genes expression and their DNA methylation status.

### Hub Genes and Tumor-Infiltrating Immune Cells and Gene Set Enrichment Analysis (GSEA)

Hub genes were uploaded to the Tumor IMmune Estimation Resource (TIMER) [37], which is a web server for comprehensive analysis of tumor-infiltrating immune cells, to study their interactions with tumor-infiltrating immune cells (B cells, CD4^+^ T cells, CD8^+^ T cells, neutrophils, macrophages and dendritic cells). Moreover, samples were divided into high-risk and low-risk groups according to the median risk score for GSEA, which was a way used to analyse and interpret coordinate pathway-level changes in transcriptomics experiments [38].

## Results

### Differential expression analysis

**Fig. 1:**
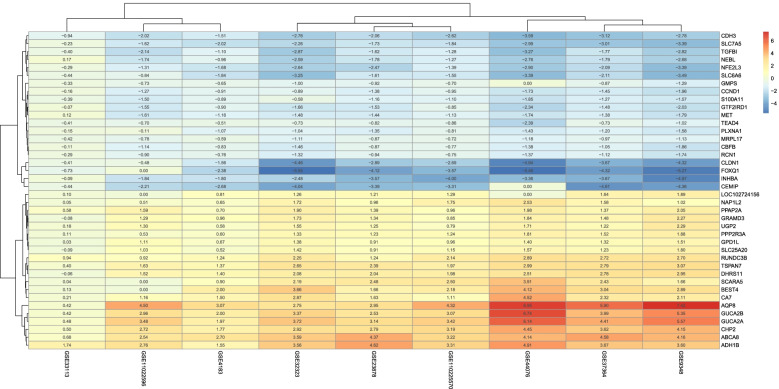
Estimation for soft-thresholding values (β). Note: Scale independence and mean connectivity varied with soft-thresholding values (β). The approximate scale-free can be attained at the soft-thresholding power of 9

DEGs were identified according to the threshold of *P*-value <0.05 based on “limma” and RRA. The top 20 down-regulated and up-regulated genes were listed based on Fig. 1. Dataset ID and 40 genes were displayed, and each gene was colored according to color key, the redder the gene, the more it was up-regulated, and the bluer the gene, the more it was down-regulated.

### Visualization of gene expression patterns and chromosome locations

**Fig. 2:**
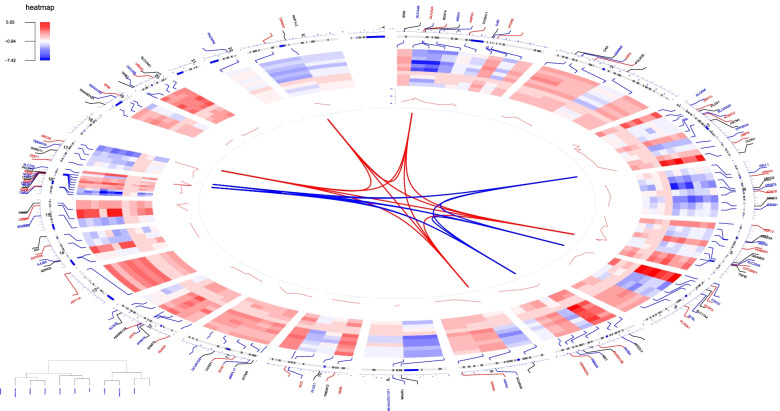
Chord plot for expression patterns and chromosomal locations of top 100 DEGs

Top 100 DEGs including top 50 up-regulated genes and top 50 down-regulated genes were preformed visualization of the expression patterns and chromosomal locations. Figure 2 illustrated that two DEGs located in chromosome X, and DEGs mainly located in chromosome 1, 3, 5 and 16. The top 5 up-regulated genes ABCA8, CLDN1, CDH3, TSPAN7 and GRAMD3 located in chromosomes 17, 3, 16, X, and 5, while top 5 down-regulated genes GRINA, PPM1H, SCD, GEMIN6 and SHMT2 located in chromosomes 8, 12, 10, 2 and 12, respectively.

### Functional annotation and visualization

**Fig. 3:**
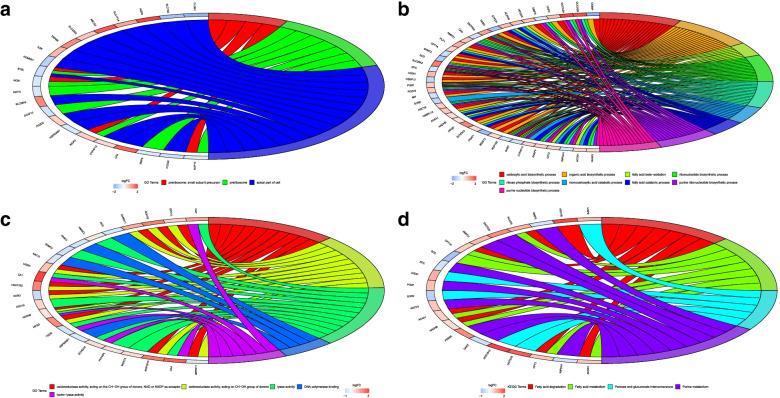
**A-D** Chord diagrams for GO and KEGG analysis of top 300 DEGs

The results of the top 300 DEGs enrichment pathway were visualized as chord patterns. In GO cellular components, GO terms including preribosome, small subunit precursor, preribosome and apical part of cell were obtained (Fig. 3A). In GO biological processes, GO terms such as carboxylic acid biosynthetic process, organic acid biosynthetic process and ribose phosphate biosynthetic process were enriched into (Fig. 3B). In GO molecular function, GO terms including oxidoreductase activity, acting on the CH − OH group of donors, NAD or NADP as acceptor, oxidoreductase activity, acting on CH − OH group of donors, DNA polymerase binding and hydro−lyase activity were obtained (Fig. 3C). For KEGG term, fatty acid degradation, fatty acid metabolism, pentose and glucuronate interconversions and purine metabolism were shown in Fig. 3D.

### Weighted gene co-expression network analysis

**Fig. 4:**
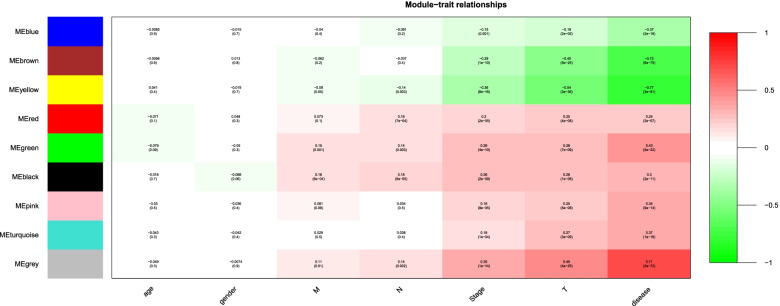
Relationships of ME and the traits such as age, gender, T, N, M, stage, disaese. Note: The X-axis showed the traits such as age, gender, T, N, M, stage, disease, the Y-axis displayed module names. Color key was displayed in the right. The rows are colored based on the correlation of the module to traits: red for positive correlation and green for negative correlation

**Fig. 5:**
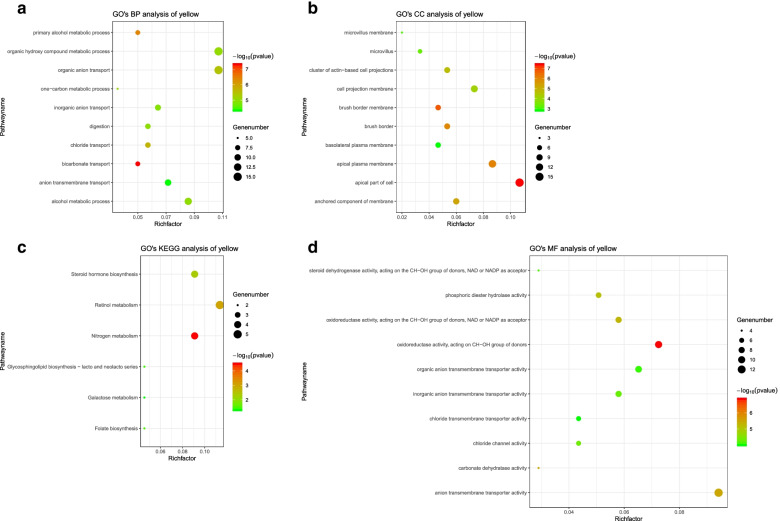
**A-D** GO terms and KEGG terms for yellow module. Note: The dot varied according to according to the catalog

After seven outliers were excluded (Supplemental Fig. 1), DEGs was used to construct a gene co-expression network based on soft threshold β = 9 (Supplemental Fig. 2). Nine gene modules were identified based on TOM and average linkage hierarchical clustering (Supplemental Fig. 3). The module had a minimum capacity of 30 genes, and non-characteristic genes were assigned to the grey module. Yellow module was identified as the interesting module for the following parameters: Yellow was significantly correlated with clinical characteristics, such as MEyellow was significantly correlated with disease (r = −0.77, *P* = 3e−91) (Fig. 4); and the module membership vs. gene significance (Supplemental Fig. 4) showed that the module was closely related to CRC (cor = 0.66, *P* = 1.7e−20). Genes in yellow module were uploaded in DAVID for functional annotations. Restricted to GO, terms such as bicarbonate transport, apical part of cell, brush border membrane and oxidoreductase activity, acting on CH − OH group of donors were obtained. Restricted to KEGG, genes were enriched in nitrogen metabolism, retinol metabolism and steroid hormone biosynthesis. The major enriched GO terms and KEGG terms associated with yellow module were illustrated in Fig. 5A-D.

### Identification, validation and efficacy evaluation for hub genes

**Fig. 6:**
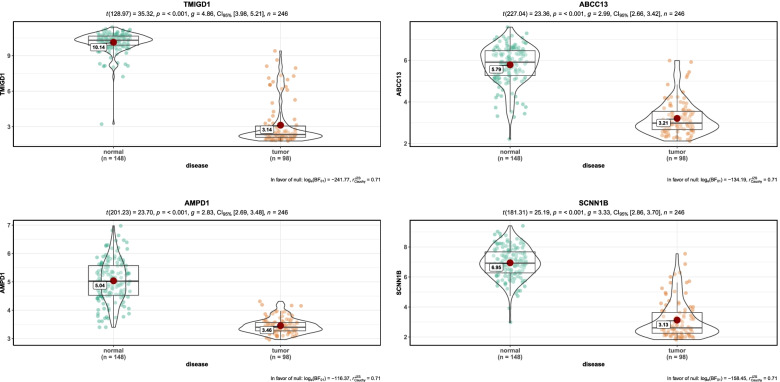
The mRNA expression of 4 top hub genes in GSE44076. Note: 4 top genes significantly down-regulated in tumor (*P* < 0.001)

**Fig. 7:**
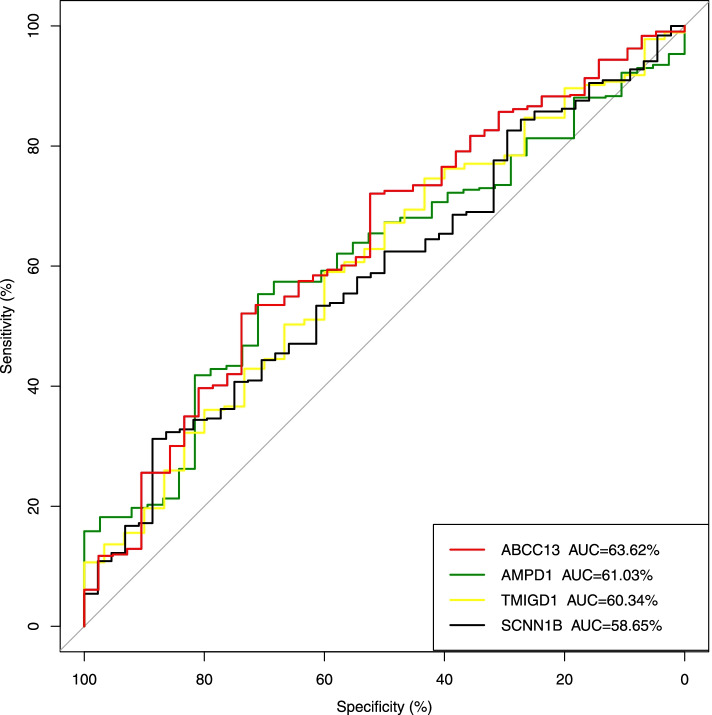
AUC of 4 top hub genes. Note: Each of the genes has an AUC of more than 50%, suggesting that these four genes may have some diagnostic value, even if with limited accuracy. The relevant data and color card were in the lower right corner

Twenty-two genes were identified when the threshold for hub genes was set as geneModuleMembership >0.80 and gene significance >0.20. In this study, we selected 4 genes less concerned by researchers as the top hub genes for further research. They were ABCC13, AMPD1, SCNN1B and TMIGD1, and all of these were down-regulated. The dataset GSE44076 was used for validation (Fig. 6), while the validation results for other eight datasets were tabulated (Table 2). In addition, AUC = 0.5–0.7 indicated that the hub gene has some diagnostic value but may have not high diagnostic accuracy (Fig. 7).

**Table 2:** Validation of 4 hub genes

Dataset ID	AMPD1	SCNN1B	ABCC13	TMIGD1
GSE4183	*P* < 0.001	*P* = 0.305	*P* = 0.006	NA
GSE44076	*P* < 0.001	*P* < 0.001	*P* < 0.001	*P* < 0.001
GSE23878	*P* < 0.001	*P* < 0.001	*P* < 0.001	NA
GSE32323	*P* < 0.001	*P* < 0.001	*P* < 0.001	NA
GSE110223	*P* < 0.001	*P* < 0.001	NA	NA
GSE110224	*P* < 0.001	*P* = 0.001	*P* < 0.001	NA
GSE33113	*P* < 0.001	*P* < 0.001	*P* = 0.006	NA
GSE37364	*P* < 0.001	*P* < 0.001	*P* < 0.001	NA
GSE9348	*P* < 0.001	*P* < 0.001	*P* < 0.001	NA

Note: Since the GPL570, GPL13667 and GPL96 platforms do not have some hub genes, the verification results of some hub genes do not exist. *P* < 0.001 indicated that these hub genes were also significantly down-regulated in these datasets

### Kaplan-Meier survival analysis

**Fig. 8:**
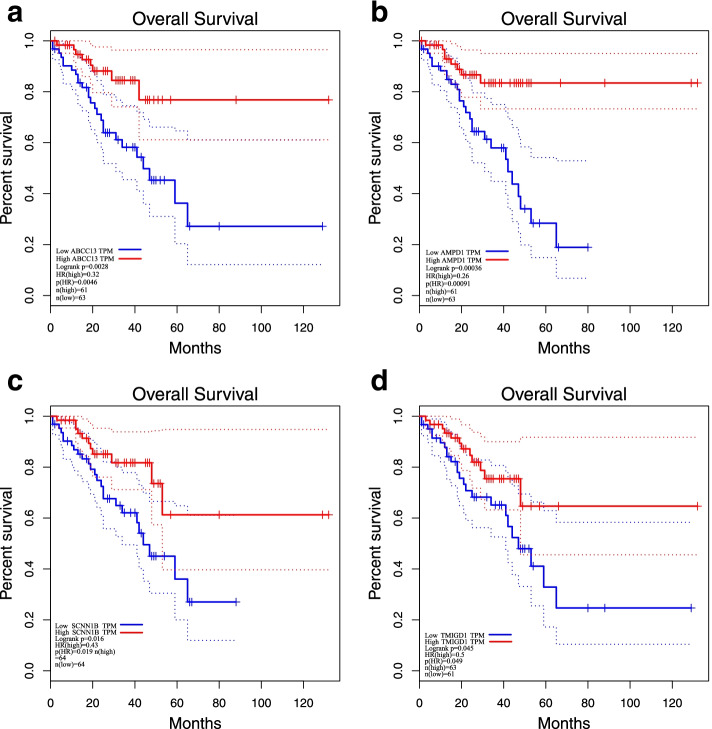
**A-D** Survival curve for testing top hub genes in GEPIA. Note: Percent survival was showed on the Y-axis, survival time (months) on the X axis. Blue represented the low-expression group, and red represented the high-expression group. This graph suggested that survival was lower in the low-expression group

All patients were divided into two groups (high versus low) according to the median expression value of 4 top hub genes and the Kaplan-Meier survival curves were plotted. The two survival curves decreased gradually with the increase of time, indicating that the survival rate decreased in both the high-expression group and the low-expression group, but the overall survival rate decreased more significantly in the low-expression group. *P*-value <0.05 was considered significant in log-rank test. Kaplan-Meier survival curves for 4 top hub genes were illustrated in Fig. 8A-D.

### DNA methylation analysis of hub genes

**Fig. 9:**
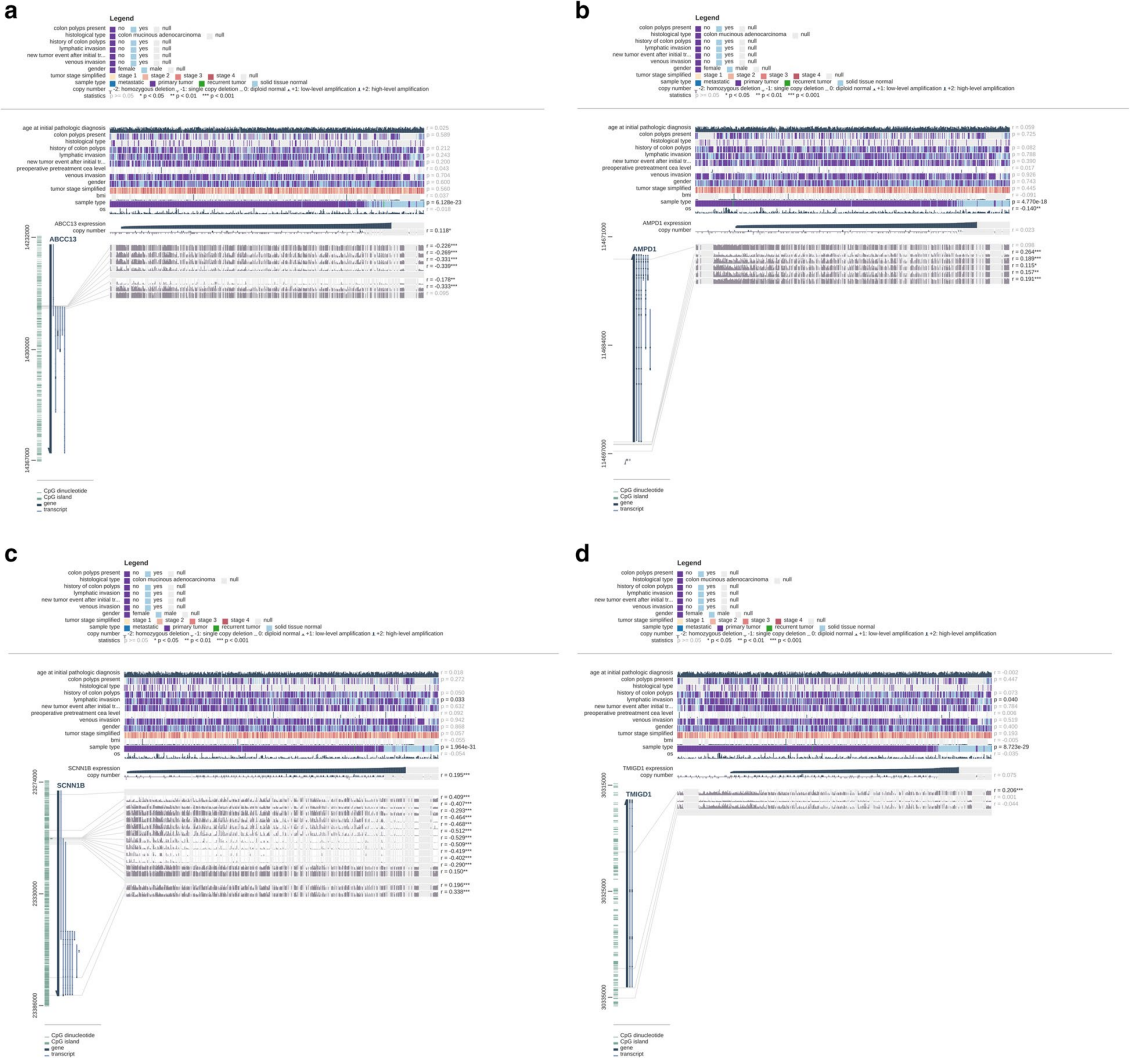
**A-D** The relationship between gene methylation and gene expression level based on colon adenocarcinoma (COAD)

**Fig. 10:**
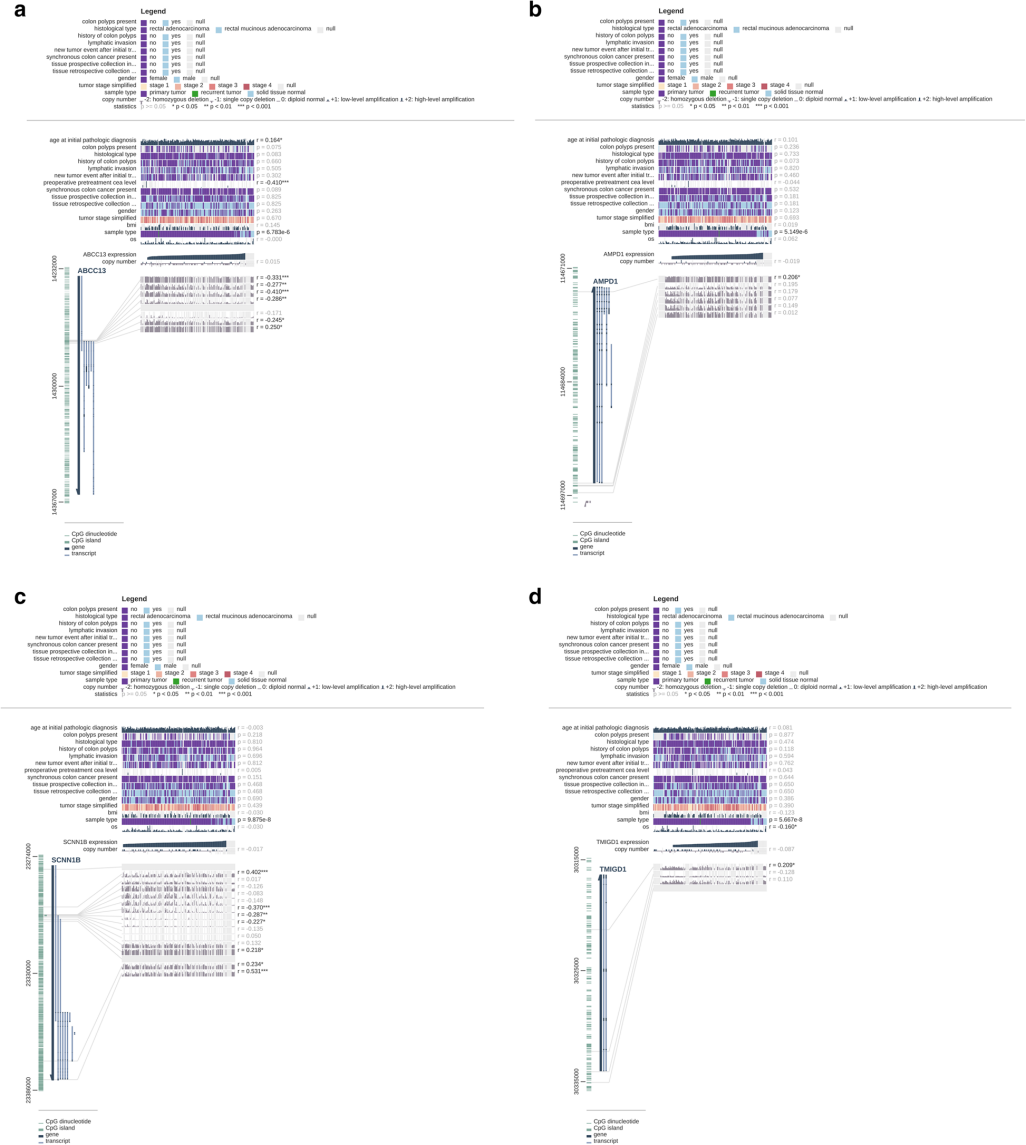
**A-D** The relationship between gene methylation and gene expression level based on rectum adenocarcinoma (READ)

DNA methylation analysis of 4 top hub genes was conducted to expound potential mechanisms of abnormal down-regulation, and methylation of these 4 genes may be used as potential biomarkers for early detection, prognosis and prediction to therapy of CRC. Supplemental Fig. 5A-D (colon adenocarcinoma) and Supplemental Fig. 6A-D (rectum adenocarcinoma) illustrated that 4 top hub genes were defined as differentially methylated genes (DMGs) with *P*-value <0.05. MEXPRESS suggests that down-regulation of some top hub genes may be caused by hypermethylation in Fig. 9A-D and Fig. 10A-D.

### Hub genes, tumor-infiltrating immune cells and GSEA

**Fig. 11:**
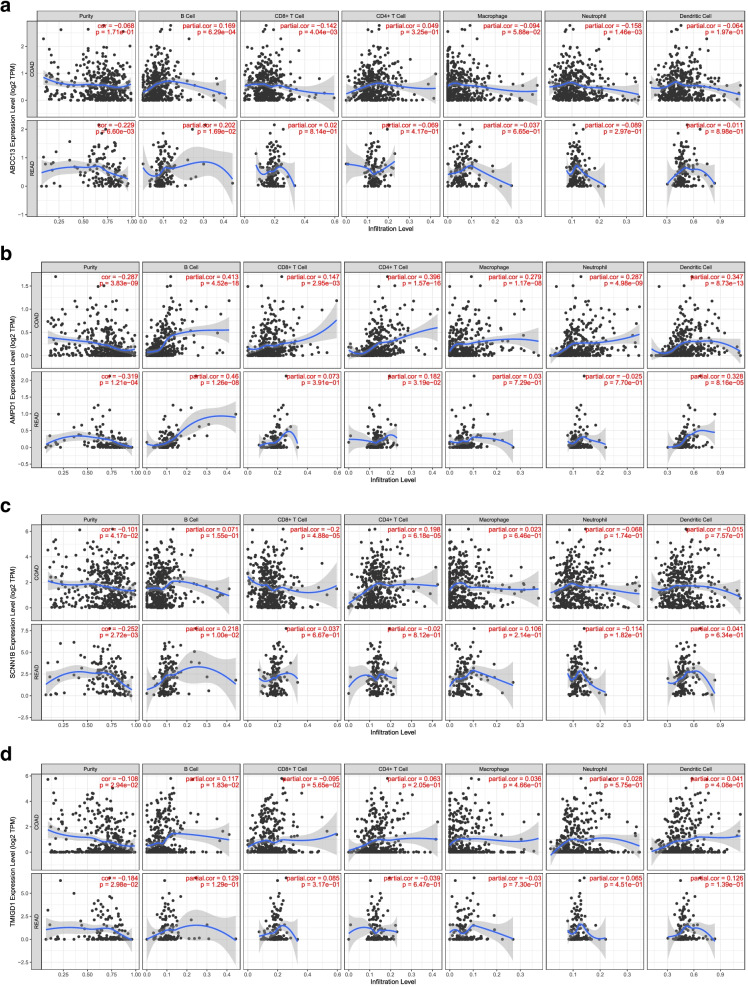
**A-D** The relationships between 4 top hub genes and tumor-infiltrating immune cells including B cells, CD4^+^ T cells, CD8^+^ T cells, neutrophils, macrophages, and dendritic cells

**Fig. 12:**
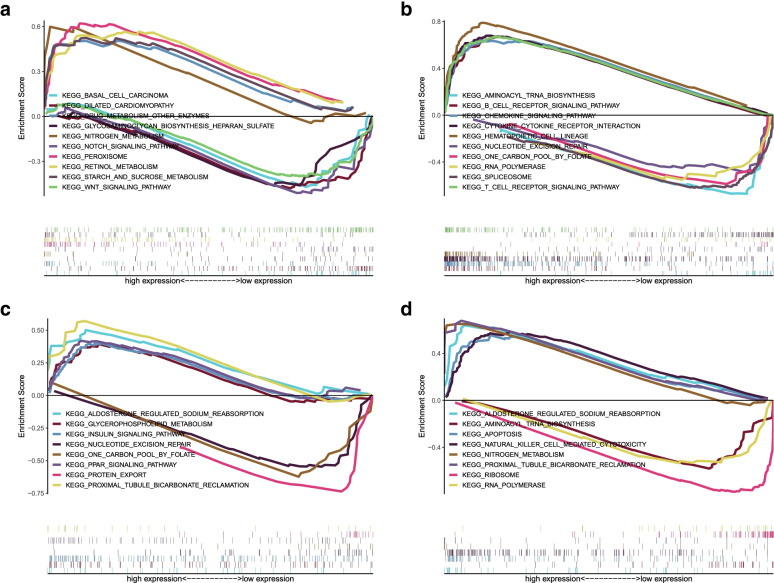
**A-D** GESA for 4 top hub genes

The relationships between 4 top hub genes and tumor-infiltrating immune cells including B cells, CD4^+^ T cells, CD8^+^ T cells, neutrophils, macrophages, and dendritic cells were carried out based on TIMER. Figure 11A-D illustrated that a relationship between top hub genes and tumor-infiltrating immune cells. The results of GESA for 4 top hub genes suggested that these pathways have a causal relationship with disease (Fig. 12A-D).

## Discussion

In this study, nine CRC datasets from the GEO were used to identify hub genes closely related to CRC based on RRA and WGCNA. Top 100 DEGs including top 50 up-regulated genes and top 50 down-regulated genes were mainly located in chromosome 1, 3, 5 and 16 based on visualization of the expression patterns and chromosomal locations in “OmicCircos” of R package. Chord patterns for top 300 DEGs illustrated that terms such as fatty acid degradation, fatty acid metabolism, carboxylic acid biosynthetic process, apical part of cell, oxidoreductase activity, acting on the CH − OH group of donors, NAD or NADP as acceptor, small subunit precursor were obtained. The genes in fatty acid metabolism were down-regulated reported, which might lead abnormal fatty acid degradation. Yellow module was a prominent module in the co-expression network constructed from TCGA samples. The MEyellow was significant correlation with disease. Restricted to GO and KEGG, genes in yellow module were mainly enriched into bicarbonate transport, apical part of cell, nitrogen metabolism and oxidoreductase activity, acting on CH − OH group of donors. These pathways suggested that these genes were involved in the development of CRC.

The more general role of DNA methylation in genome stability can be achieved by chromatin structure modeling, which is the main role of methyl groups [39]. Although the exact mechanism by which DNA methylation affects chromatin structure remains unclear, sequence independent methyl parts play a direct role in the production of closed chromatin structure [40]. DNA methylation may form chromatin and gene expression states through internal effects on nucleosome structure and/or by regulating other factors that replace nucleosomes [41]. The promoter CpG island of active genes in normal (or cancer) cells is characterized by open chromatin region, lack of DNA methylation, nucleosome deletion (detected by hypersensitive sites) and histone posttranslational modifications, which are typical features of active genes [42]. Open chromatin structures that determine the expression status of active genes may increase the possibility of DNA damage and may disrupt enzyme trading. Privitera’s study [43] disclosed that transcriptome comparisons among the 1,16-chromogroups for breast cancer, integrated with functional pathway analysis, suggested the cooperation of overexpressed 1q genes and underexpressed 16q genes in the genesis of both ductal and lobular carcinomas, thus highlighting the putative role of genes encoding gamma-secretase subunits and WNT enhanceosome components in 1q, and the glycoprotein E-cadherin, the E3 ubiquitin-protein ligase WW domain-containing protein 2, the deubiquitinating enzyme CYLD, and the transcription factor core-binding factor subunit beta in 16q. The analysis of 1, 16-chromogroups is a strategy with far-reaching implications for the selection of cancer cell models and novel experimental therapies. Detection of complex cytogenetic abnormalities (3 abnormalities), hypodiploidy, monosomy 13/del(13q) or monosomy 17/del(17p) on conventional cytogenetics in a patient with multiple myeloma should be considered as indicative of a more adverse prognosis [44]. A better understanding of the significance of DNA methylation machinery and chromatin structure in maintaining genome integrity will facilitate future investigations to target DNA methylation and its mediators for novel drugs and chemotherapeutic combinations.

Yellow module, which stood out because of its significant correlation with clinical traits, participated in the follow-up analysis as a key module. 4 significantly down-regulated DEGs ABCC13, AMPD1, SCNN1B and TMIGD1 were identified as top hub genes in yellow module. Nine data sets from GEO were used to validate the genes: the hub genes were almost all significantly down-regulated in these nine data sets. In addition, to evaluate the efficacy of hub genes, the ROC curve was constructed and the AUC made a summary: the hub gene had some diagnostic value. Kaplan-Meier Survival Analysis of the four hub genes showed that the overall survival rate of the low-expression group was lower than the high-expression group, suggesting that significant down-regulation of hub genes had an impact on the prognosis of CRC patients. There was a correlation between tumor-infiltrating immune cells (B cells, CD4^+^ T cells, CD8^+^ T cells, neutrophils, macrophages, and dendritic cells) and hub genes. GSEA suggested that these genes are closely associated with CRC progression.

SCNN1B (sodium channel epithelial 1 subunit beta), a methylation-related differentially expressed gene was mentioned in gastric cancer [45] and renal cell carcinoma (RCC) [46]. Based on the analysis of tissue chips and related studies, the gene with anti-tumor function was reported that it might be a potential survival marker for gastric cancer [47]. SCNN1B overexpression was sufficient to suppress multiple features of cancer cell pathophysiology in vitro and in vivo Mechanistic investigations revealed that SCNN1B interacted with the endoplasmic reticulum chaperone, GRP78, and induced its degradation via polyubiquitination, triggering the unfolded protein response (UPR) via activation of PERK, ATF4, XBP1s, and C/EBP homologous protein and leading in turn to caspase-dependent apoptosis [47]. This gene was one of the genes with RCC-specific promoter methylation and down-regulation [48]. A comprehensive analysis of RCC molecular subtypes defined by specific promoter methylation (including SCNN1B) showed that dietary intakes are differentially associated with ccRCC risk [48]. Remarkably, MEXPRESS suggested that the significantly low expression of this gene may be related to hypermethylation in the study. However, the specific underlying mechanism of this gene in CRC is still unclear. The researchers paid less attention to the other three top hub genes than to this one. To our knowledge, the transmembrane and immunoglobulin domain containing 1 (TMIGD1) may be associated with intestinal differentiation and was considered a tumor suppressor (TMIGD1 significantly down-regulated in renal cancer) [49]. In addition, the loss of TMIGD1 significantly impaired intestinal epithelium brush border membrane, junctional polarity, and maturation. Mechanistically, TMIGD1 inhibits tumor cell proliferation and cell migration, arrests cell cycle at the G2/M phase, and induces expression of p^21CIP1^ (cyclin-dependent kinase inhibitor 1), and p^27KIP1^ (cyclin-dependent kinase inhibitor 1B) expression, key cell cycle inhibitor proteins involved in the regulation of the cell cycle [50]. Moreover, TMIGD1 is shown to be progressively down-regulated in sporadic human CRC, and its downregulation correlates with poor overall survival. The findings herein identify TMIGD1 as a novel tumor suppressor gene and provide new insights into the pathogenesis of colorectal cancer and a novel potential therapeutic target [50]. AMPD1 played an important role in the purine nucleotide cycle, and ABCC13 may be an important agent of drug resistance [51]. Related studies [52] had confirmed the expression level of AMPD gene in tumors and healthy livers. The explanation of the augmented activity of AMP-deaminase in the tumor tissue may be related with changed resistance of the enzyme toward proteolytic action of intracellular proteases. A quite opposite may be true in the case of AMP-deaminase isolated from cirrhotic liver [53]. Here, diminished resistance of the enzyme for intracellular proteolysis has been suggested as a probable factor diminishing activity of AMP-deaminase [54]. However, no evidence has been reported on the role and mechanism of ABCC13 gene in cancer. Further attention needs to be paid to the specific biological mechanism between the occurrence and development of CRC in these 4 top hub genes.

## Conclusions

In summary, four DEGs were identified in the yellow module as top hub genes that strongly correlated with CRC, and that significantly low expression of hub genes led to poor prognosis. The significant down-regulation of some genes may be related to hypermethylation. In addition to the correlation between tumor-infiltrating immune cells and the degree of down-regulation of genes, there was a close relationship between hub genes and the progression of CRC. The study may provide a little new insights and directions into the potential pathogenesis of CRC.

## Supplementary Information

**Additional file 1: Supplemental Figure 1.** Sample dendrogram and trait heatmap. **Supplemental Figure 2.** Heat map for top 20 down-regulated and top 20 up-regulated genes. Note: The gene symbols were listed in Y-axis and the datasets were listed in X-axis. Color-coded according to correlation coefficient (legend at right). **Supplemental Figure 3.** Cluster dendrogram. Note: Module identification is based on gene expression similarity. The genes with similar expression clustered according to a topological overlap metric into modules; assigned modules were colored and uncharacteristic genes assigned to gray module. **Supplemental Figure 4.** The scatter diagram between the module membership in yellow module and gene significance for disease. **Supplemental Figure 5.** A-D DNA methylation of 4 top hub genes based on colon adenocarcinoma (COAD). **Supplemental Figure 6.** A-D DNA methylation of 4 top hub genes based on rectum adenocarcinoma (READ).

## Data Availability

The data that support the findings of this study are openly available in GEO (https://www.ncbi.nlm.nih.gov/geo/).
