# Supplementary material for: Identification of four novel hub genes as monitoring biomarkers for colorectal cancer
Source: Hereditas. 2022 Jan 29;159:11. doi: 10.1186/s41065-021-00216-7 (PMC8801129; doi:10.1186/s41065-021-00216-7)

**Legend of Supplemental Materials**

**Supplemental Figure 1** Sample dendrogram and trait heatmap.

**Supplemental Figure 2** Heat map for top 20 down-regulated and top 20 up-regulated genes.

**Note:** The gene symbols were listed in Y-axis and the datasets were listed in X-axis. Color-coded according to correlation coefficient (legend at right).

**Supplemental Figure 3** Cluster dendrogram.

**Note:** Module identification is based on gene expression similarity. The genes with similar expression clustered according to a topological overlap metric into modules; assigned modules were colored and uncharacteristic genes assigned to gray module.

**Supplemental Figure 4** The scatter diagram between the module membership in yellow module and gene significance for disease

**Supplemental Figure 5A-D** DNA methylation of 4 top hub genes based on colon adenocarcinoma (COAD).

**Supplemental Figure 6A-D** DNA methylation of 4 top hub genes based on rectum adenocarcinoma (READ).

**Supplemental Figure 1** Sample dendrogram and trait heatmap.

**
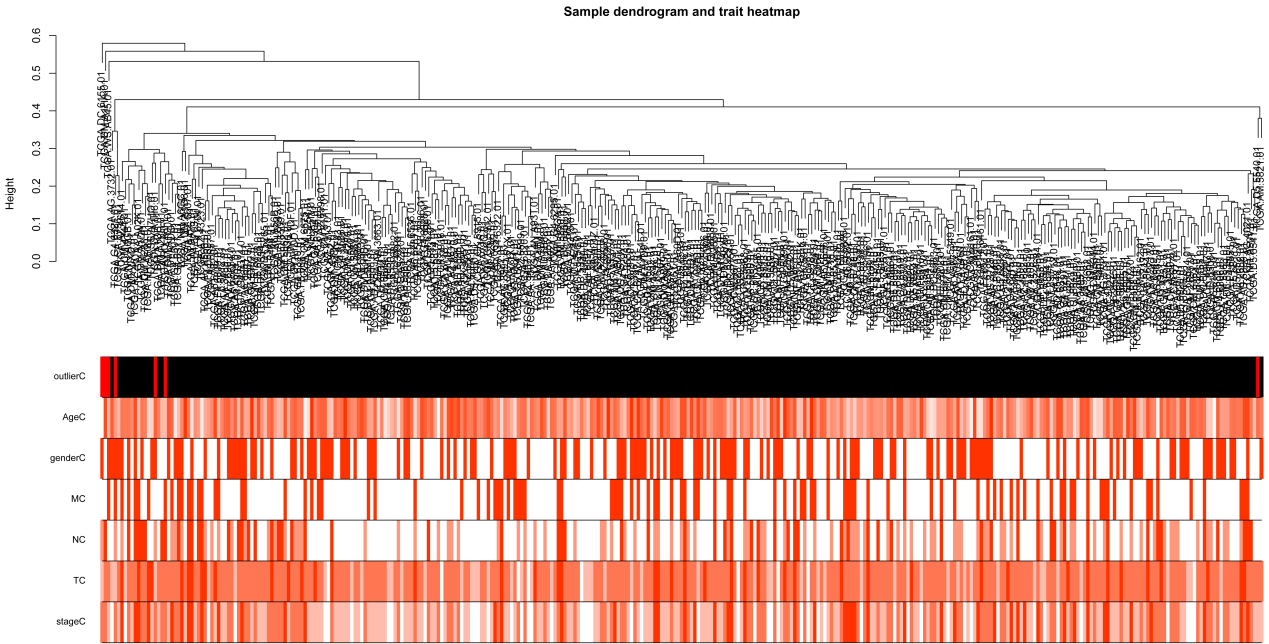
**

**Supplemental Figure 2** Heat map for top 20 down-regulated and top 20 up-regulated genes.


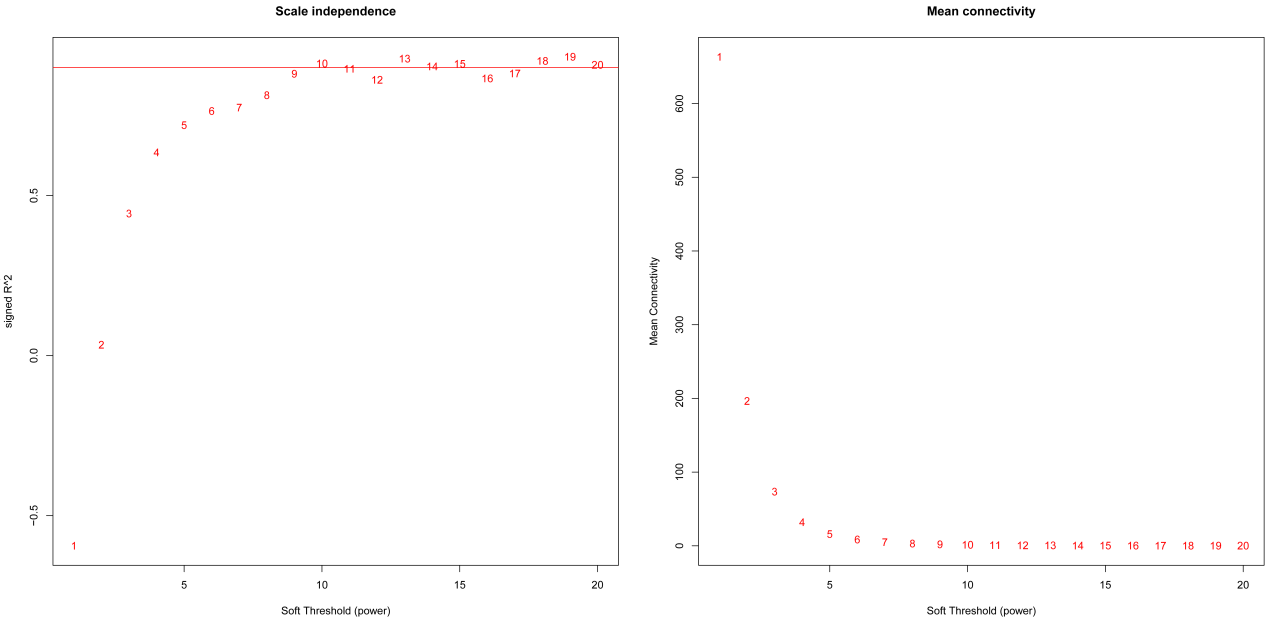


**Note:** The gene symbols were listed in Y-axis and the datasets were listed in X-axis. Color-coded according to correlation coefficient (legend at right).

**Supplemental Figure 3** Cluster dendrogram.


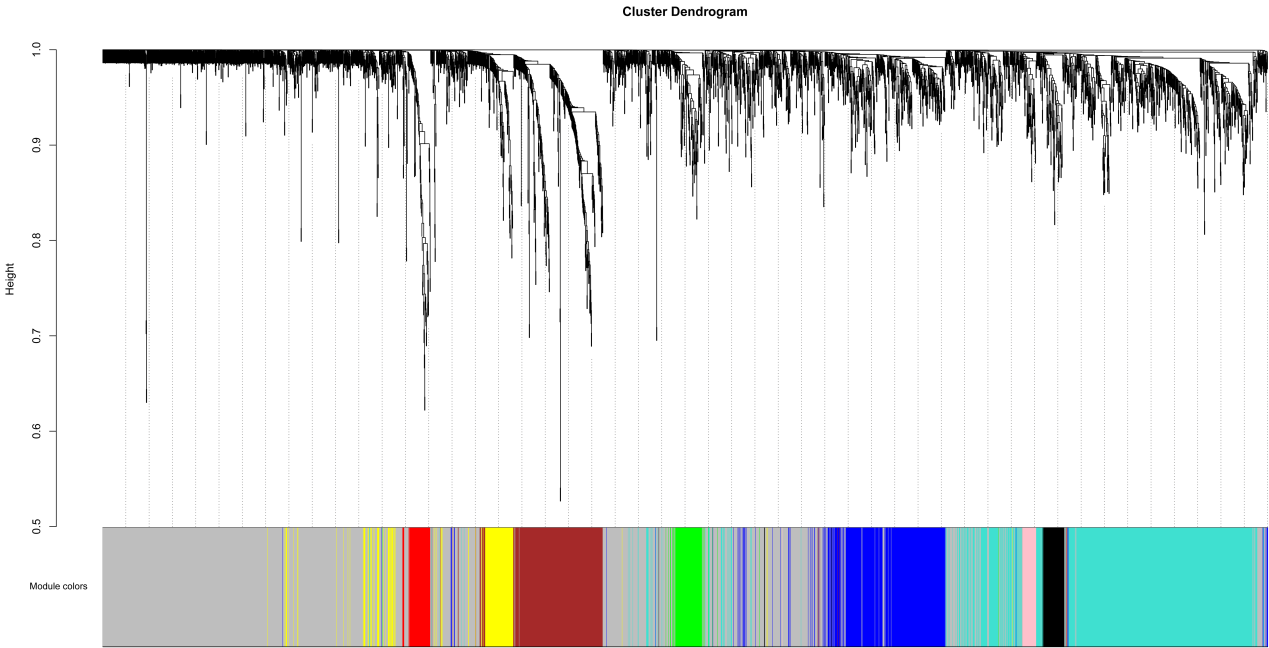


**Note:** Module identification is based on gene expression similarity. The genes with similar expression clustered according to a topological overlap metric into modules; assigned modules were colored and uncharacteristic genes assigned to gray module.

**Supplemental Figure 4** The scatter diagram between the module membership in yellow module and gene significance for disease

**
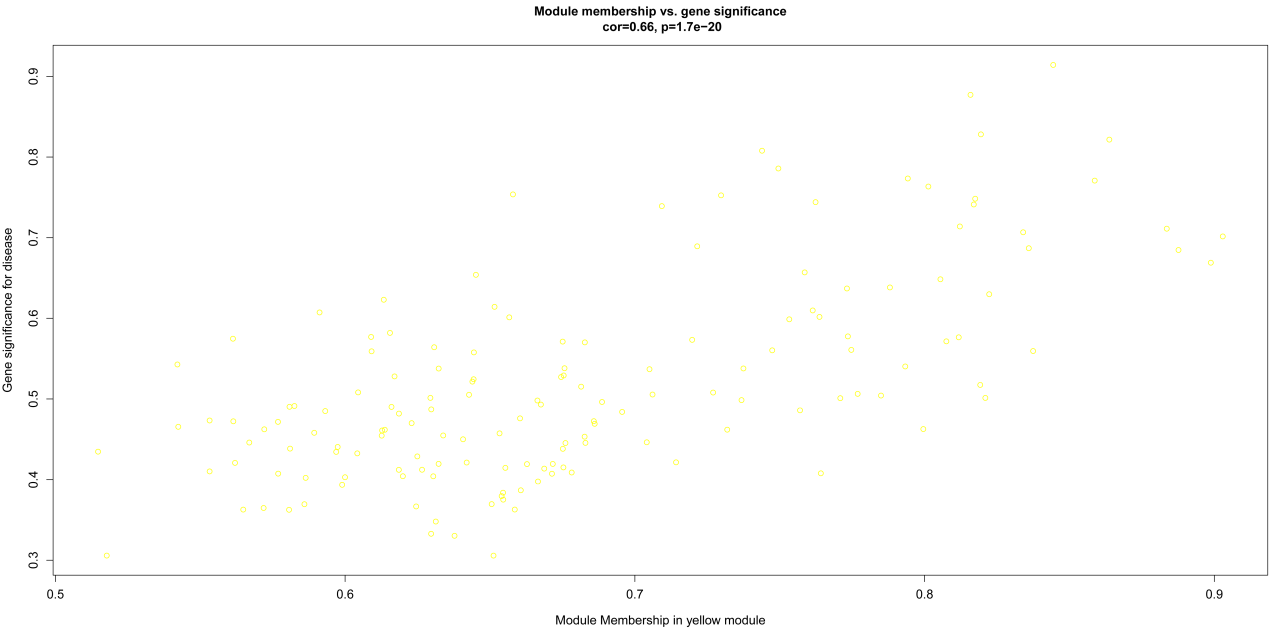
**

**Supplemental Figure 5A-D** DNA methylation of 4 top hub genes based on colon adenocarcinoma (COAD).

**Supplemental Figure 5A: ABCC13**

**
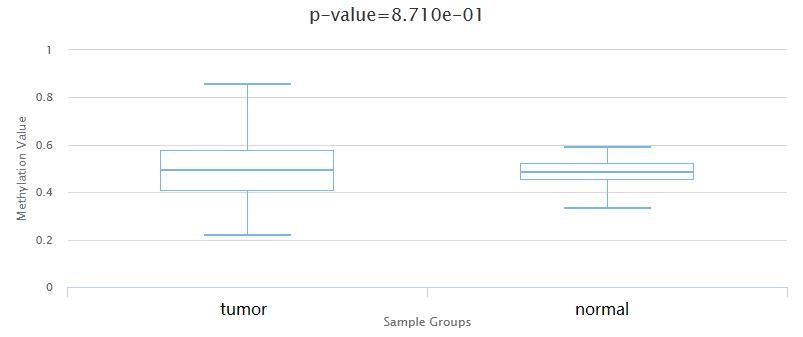
**

**Supplemental Figure 5B: AMPD1**

**
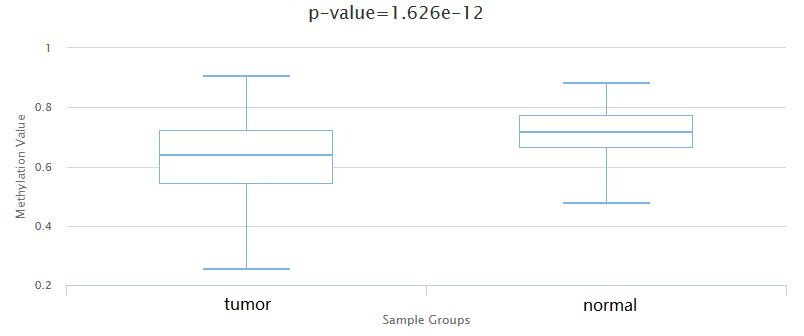
**

**Supplemental Figure 5C: SCNN1B**

**
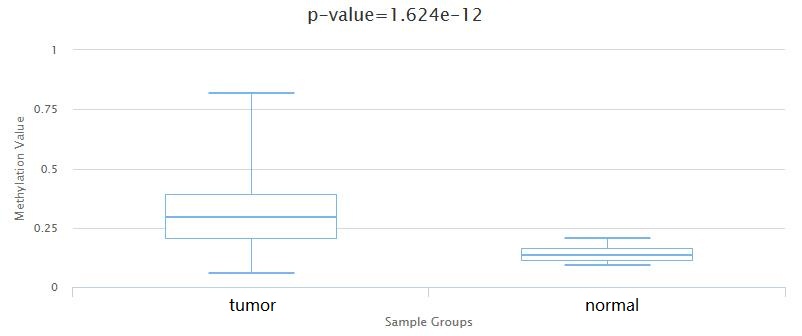
**

**Supplemental Figure 5D: TMIGD1**

**
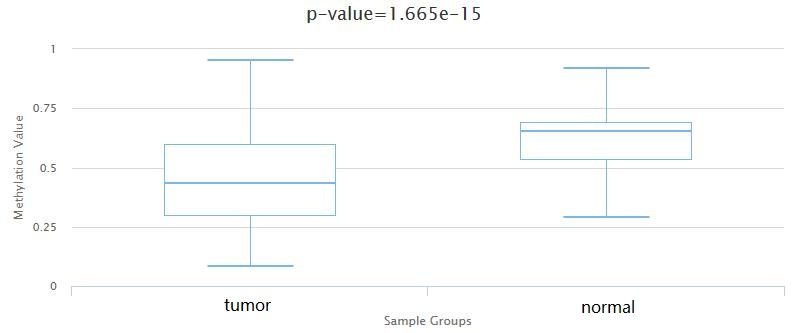
**

**Supplemental Figure 6A-D** DNA methylation of 4 top hub genes based on rectum adenocarcinoma (READ).

**Supplemental Figure 6A: ABCC13**

**
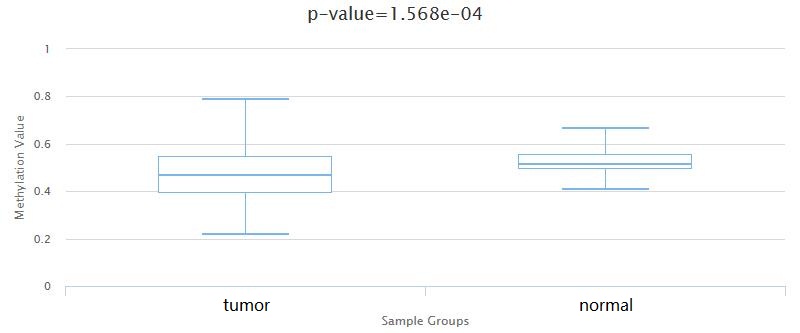
**

**Supplemental Figure 6B: AMPD1**

**
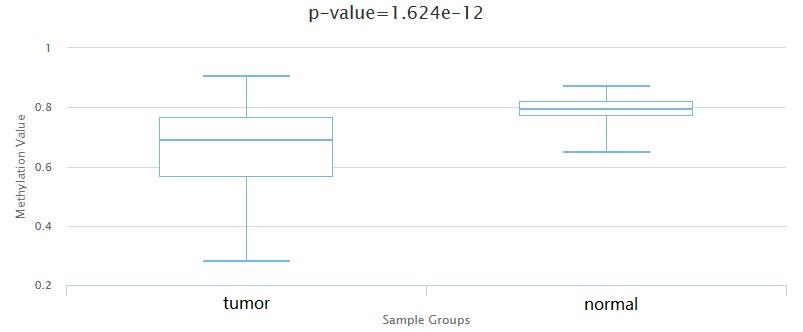
**

**Supplemental Figure 6C: SCNN1B**

**
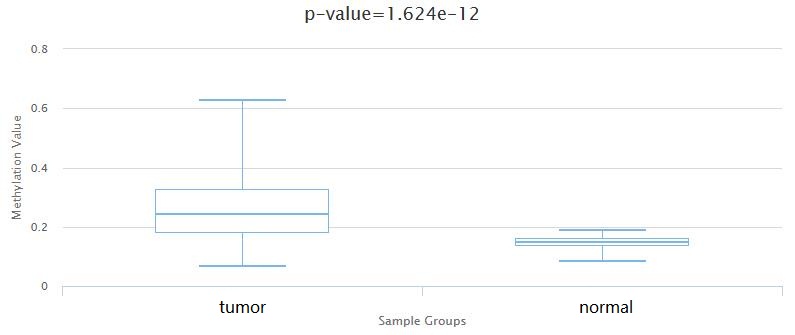
**

**Supplemental Figure 6D: TMIGD1**


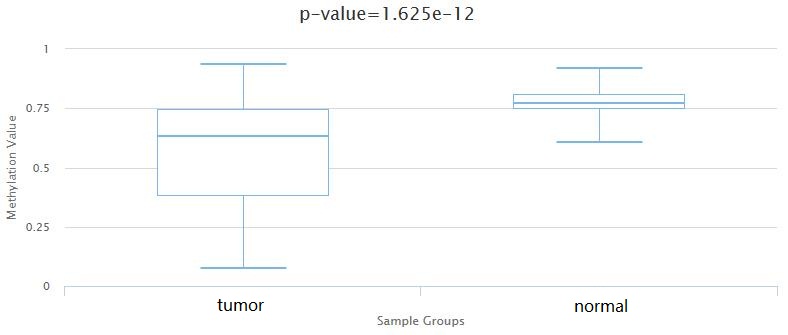

Supplement: Supplementary file 1 — Additional file 1: Supplemental Figure 1. Sample dendrogram and trait heatmap. Supplemental Figure 2. Heat map for top 20 down-regulated and top 20 up-regulated genes. Note: The gene symbols were listed in Y-axis and the datasets were listed in X-axis. Color-coded according to correlation coefficient (legend at right). Supplemental Figure 3. Cluster dendrogram. Note: Module identification is based on gene expression similarity. The genes with similar expression clustered according to a topological overlap metric into modules; assigned modules were colored and uncharacteristic genes assigned to gray module. Supplemental Figure 4. The scatter diagram between the module membership in yellow module and gene significance for disease. Supplemental Figure 5. A-D DNA methylation of 4 top hub genes based on colon adenocarcinoma (COAD). Supplemental Figure 6. A-D DNA methylation of 4 top hub genes based on rectum adenocarcinoma (READ). [file 41065_2021_216_MOESM1_ESM.docx]
